# Evaluation of Compressive and Bending Strength of a Geopolymer Based on Lateritic Clays as an Alternative Hydraulic Binder

**DOI:** 10.3390/ma17020307

**Published:** 2024-01-08

**Authors:** Walter A. Abujder Ochoa, Moisés A. Sánchez Málaga, Arturo Brañez Tapia, Oriana Palma Calabokis, Yamid E. Nuñez de la Rosa, Gunther E. Viscarra Chirinos, Sebastián N. Pinto Lavayén

**Affiliations:** 1 Universidad Católica Boliviana San Pablo, Departamento de Ingenierías y Ciencias Exactas, Centro de Investigación en Ciencias Exactas e Ingenierías (CICEI), C. Márquez, Esq. Parque Jorge Trigo Andia, Tupuraya, Cochabamba, Bolivia; moises.sanchez@ucb.edu.bo (M.A.S.M.); arturo.branez@ucb.edu.bo (A.B.T.); gviscarra@ucb.edu.bo (G.E.V.C.); sebastian.pinto@ucb.edu.bo (S.N.P.L.); 2 Faculty of Engineering and Basic Sciences, Fundación Universitaria Los Libertadores, Bogotá 1112211, Colombia; yenunezd@libertadores.edu.co

**Keywords:** lateritic soil, geopolymer, Portland cement, bending strength, compressive strength

## Abstract

In Bolivia, lateritic soils are common in humid tropical regions and can be used in the construction industry as an alternative to materials that cause a negative environmental impact, such as cement. The production of Portland cement causes environmental issues like significant greenhouse gas emissions and air pollution. To address this problem, geopolymers have been introduced as an alternative binder with low CO_2_ emissions. In this regard, geopolymers based on lateritic clays have been studied mineralogically, chemically, and on their compressive strength separately. However, there are still no studies on lateritic clays present in Bolivia and their mechanical, mineralogical, and chemical properties combined in a geopolymer. Therefore, this present research proposes the evaluation of a geopolymer made from laterite clays. Compression and flexural tests were carried out, along with mineralogical and chemical analyses on mortar and geopolymer cubes and prisms. The results indicate that the laterite clay-based geopolymer has lower compressive strength compared to Portland cement IP (cement type I with the addition of pozzolana) mortar. However, the flexural strength tests show a slight increase in the case of the geopolymer.

## 1. Introduction

Various pollutants cause damage to the environment and, therefore, dangerous consequences for humans, animals, and plants due to their accumulation in the environment. These pollutants are of great concern worldwide, so several studies are being conducted to develop new ways to reduce them [1]. In recent years, different industries have sought to reduce their environmental impact by innovating products that do not cause environmental problems. The construction sector has been no exception. Nevertheless, cement continues to be one of the materials that generates the most pollution in its manufacturing process. However, it is still one of the most frequently used materials in the construction industry.

It is indisputable that the cement industry is currently facing a significant identity crisis, with key figures (architects, engineers, and even a portion of the scientific community) being fully aware of this reality [2]. On one hand, the economic progress of developing countries, an inconceivable process without the rapid construction of modern and extensive infrastructures, clearly foresees a substantial increase in cement demand for the next 40–50 years, potentially doubling or even tripling current production. However, on the other hand, the existing technology in Portland cement, a legacy of the 20th century characterized by high energy consumption, the use of non-renewable resources, substantial carbon dioxide emissions, and questionable durability, among other aspects, is unequivocally incompatible with a rational notion of ‘sustainable’ development [2].

The literature and research suggesting an immediate change in construction binder technology argue that a new product cannot be launched into the market without undergoing exhaustive scientific-technical analysis. The scientific history of these materials presents inherent complexity and a considerable level of difficulty. Firstly, a single physicochemical concept has given rise to diverse terminology: terms such as alkaline cements, geocements, geopolymers, and inorganic polymers are used to describe the alkaline activation of materials such as kaolin, limestone, or dolomite [2].

Concrete accounts for 5–8% of total anthropogenic CO_2_ emissions, with 95% of CO_2_ produced during cement manufacturing. Half of it is released due to limestone decarbonization during cement production. However, due to consumption growth, especially in developing countries, it is challenging to envision a reduction in CO_2_. Hence, alternative cements are urgently needed. They are commonly referred to as geopolymers. Nevertheless, it is important to note that not all geopolymers exhibit a low carbon footprint; only a specific subset of geopolymers, termed ‘one-part geopolymers’, has demonstrated significantly lower carbon footprint levels than Portland cement-based mixtures. Alkali-activated cements and concretes are another promising alternative, formed by the alkali activation of aluminosilicate sources, which can be natural materials like lateritic clays, synthetics, or industrial wastes [3].

Although the use of Portland cement is frequent, it is recognized that its production leads to many environmental problems, such as the depletion of natural resources, environmental degradation, high greenhouse gas emissions, and atmospheric pollution. The significant emission of greenhouse gases in cement manufacture is considered a significant contribution to global warming [4]. In this regard, in order to find a solution that does not have a negative environmental impact, an alternative binder with low CO_2_ emissions was implemented, such as a geopolymer [5]. This material is an alternative to Portland cement and has several advantages from an environmental perspective, as well as high compressive strength and durability against chemical attack [6,7]. Generally, conventional geopolymer comprises precursor materials with a high proportion of aluminosilicate, such as fly ash, and aluminosilicate [8], bottom ash, slag [9,10], and ashes from the incineration of municipal solid wastes [11]. The alkaline activator for conventional geopolymers is usually a combination of two chemical compounds: sodium silicate (NS) and sodium hydroxide (NH). A wet amalgamation process usually prepares the mixture.

Although fly ash-based geopolymers have been studied as a possible alternative in recent years, they have yet to become a viable option due to the complexity of obtaining the material and the cost involved [12]. In this sense, lateritic clays form in humid tropical regions characterized by warm, rainy climates where sedimentary materials resulting from rock decomposition are deposited. These soils exhibit specific properties rendering them valuable for both construction and agricultural purposes. In Bolivia, the eastern regions host the development of lateritic soils owing to a range of favorable conditions during different phases of their formation and alteration.

However, this specific Bolivian region lacks materials meeting the stringent specifications mandated by regulations. Additionally, transportation expenses associated with these materials significantly escalate the construction costs. Consequently, exists a necessity to conduct studies for lateritic soils. This examination aims to elucidate technical concepts ensuring their optimal utilization, not solely restricted to road constructions, but extending to a diverse application in civil constructions.

Laterites are reddish and yellowish soils formed in tropical and subtropical areas. They are formed due to kaolinite alteration by iron minerals through the induration phenomenon. Lateritic clays are a material that comes from the lateritic soil typical of tropical and subtropical regions, typical of the Bolivian pre-Cambrian, ranging from the province of San Ramon, which is located in the city of Santa Cruz and continues through the department of Beni until reaching Brazil [13].

Rodrigue et al. compiles all the research carried out on laterite and its use as a geopolymer, but the performance of the binder was evaluated in terms of resistance only on compressive strength [5]. Kaze et al. also examined how the silica modulus in the activating solution affects the performance of iron-rich laterite-based geopolymer binders subjected to calcination at 600 °C [14]. This research indicated that a silica modulus 0.75 was the optimum for achieving the highest compressive strength in calcined laterite; however, the study focuses solely on the alkaline activation of iron-rich aluminosilicates (laterites), without conducting additional strength or flexural tests to evaluate the geopolymer. On the other hand, Poudeu et al. combined crude and calcined laterite (at temperatures between 500–600 °C) to produce geopolymeric materials based on Cameroonian lateritic soil and found that the synthesized products could be a low-cost alternative for housing construction [15]. Similarly, Kamseu et al. used iron-rich laterite in its raw state and reactive silica from rice husk ash as starting materials for geopolymer production. The results demonstrated the formation of new bonding phases, such as hinsingerite. This additional formation enhanced the geopolymer binder, resulting in improved cohesion between particles and increased densification of the interconnected pores in the matrix [16].

These studies collectively highlight the importance of lateritic clay-based geopolymer. However, mineralogical, chemical, and mechanical property studies have not been carried out in conjunction, in order to study the effectiveness of the geopolymer and its application in construction, beyond only applying them to roads. Therefore, by reviewing the existing materials that can be used in Bolivia, the present research proposes to evaluate a geopolymer based on laterite clays, from its mechanical properties of compression and bending, its mineralogical and chemical composition, as an alternative to hydraulic conglomerate that would significantly reduce the environmental impact of its manufacturing process, taking into account that in Bolivia the environmental impact of the cement manufacturing process is high. To this end, the evaluation of the geopolymer based on lateritic clays was carried out through the elaboration of cubes and prisms that were subjected to mechanical tests of compression, bending, chemical, and mineralogical studies.

Regarding the results of the present investigation, the values of the average resistance of the compression tests of the samples of Portland cement IP, a hydraulic cement in which the constituent pozzolan is present up to 40% by mass, and the geopolymer indicate that the geopolymer based on laterite clay shows a significant difference of lower resistance with the Portland cement mortar. Likewise, the average flexural strength tests of the most representative samples of the geopolymer indicate a slight increase concerning the prismatic cement mortar specimens, which generates expectations regarding the different uses that can be given to the evaluated geopolymer, thus opening new lines of research.

## 2. Experimental Program

### 2.1. Materials

Lateritic soils are typical of tropical areas and cover an extensive strip from the province of San Ramón located in the department of Santa Cruz, Bolivia, which continues through the department of Beni—Bolivia—until reaching an extensive area of Brazil. In this sense, the material object of this study is extracted from the RN 10 route on the way to San Javier located in the department of Santa Cruz, whose coordinates of the extraction area are: Latitude 16°17′39.86″ S; Longitude 62°31′4.38″ W. The exploration of the deposit was carried out to obtain the required mineral raw material as indicated by ASTMC 75 [17].

**Figure 1:**
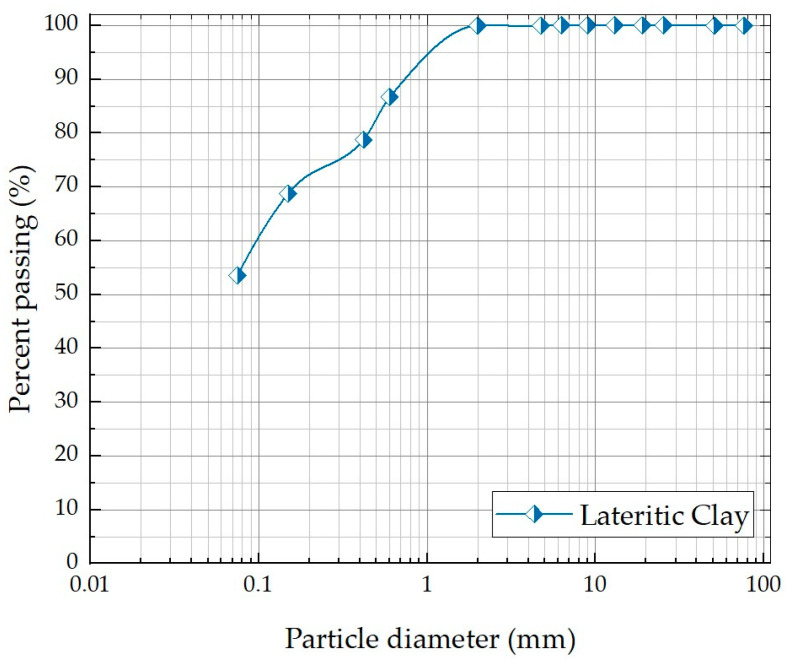
The granulometric curve of the lateritic clay.

For the testing process, sand was used to make the cement mortar, and laterite clay was used for the geopolymer. The granulometric curve of the laterite clay sample analyzed is shown in Figure 1. The high content of fines in the samples collected in this study (%) is related to the fact that, during the exploration with machines, the laterite sand layers are mixed, unintentionally, with a portion of the lower layer that is mainly composed of fine soil. The resulting material is representative of the field exploration process. Table 1 shows the physical properties of the laterite soil studied and the standards used for characterization tests. This material is classified as low plasticity clay (CL) by the Unified Soil Classification System [18,19] and classified by AASHTO [20] as clay soil (A-6 (4)).

As for the fine sieved soil, it is observed that the particles have an orange hue. When this fine material is exposed to water, it takes on a very particular reddish color, which allows to observe that this material has a high content of iron and aluminum, as well as high silica content whose contribution is of a whitish color, and the combination of both gives a characteristic orange color.

The chemical composition of the fine fraction, analyzed by photometry and X-ray fluorescence assay, determined that the sample is composed of aluminum (19.84%), iron (5.42%), sodium (0.10%), silica (40.71%), aluminum oxide (0.95), and iron oxide (20.13%). These chemical and mineralogical characteristics are similar to other studies in Brazil, India, Australia, and Africa [22,23].

Portland cement (IP type) was used for the mortar manufacturing process [24]. This cement has between 20% and 40% pozzolana in its composition. Portland cement type IP is the binder widely produced and used for construction in Bolivia. The cement has a specific gravity of 2.90.

### 2.2. Specimens Molding and Preparation

**Figure 2:**
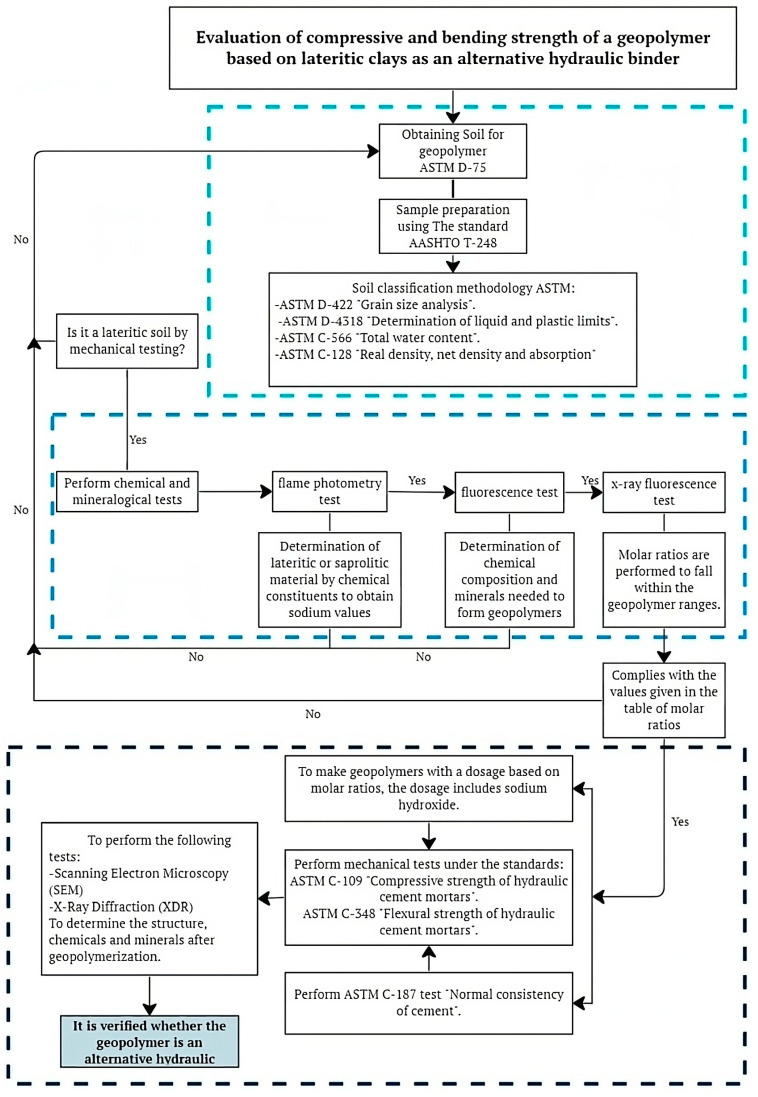
Experimental Program Flowchart.

The molding and preparation of specimens followed the detailed flowchart presented in Figure 2, designed for the comparison between two types of mortars: one based on Portland cement and the other on geopolymers, focusing on their compression and flexural strength. Material characterization, including Portland cement and sand, preceded the determination of proportions for the Portland cement-based mortar, following ASTM C109 regulations [25].

For the compression test, a mixture of 82 g of Portland cement, 226 g of sand, and 60 mL of water was used in a mold with dimensions of 50 mm × 50 mm × 50 mm. In the flexural test, the mixture consisted of 166 g of Portland cement, 458 g of sand, and 60 mL of water, in a mold with dimensions of 40 mm × 40 mm × 160 mm. Verification of the geopolymers based on lateritic clay and sodium hydroxide involved three processes. First, the material was obtained and characterized through physical tests, revealing low plasticity clay with a plasticity index of 15.96%. Subsequently, it was confirmed that the material corresponded to lateritic soil through chemical and mineralogical tests, validating the sodium content at 10%, lower than the required 15% for it to be considered saprolitic material.

Molar ratios were verified through fluorescence and X-ray tests. The final dosing for the compression test was 1 L of distilled water, 480 g of NaOH, 190 g of lateritic clay, and 118 g of sand, in a mold of dimensions 50 mm × 50 mm × 50 mm. For the flexural test, the dosing consisted of 1 L of distilled water, 480 g of NaOH, 530 g of lateritic clay, and 168 g of sand, in a mold of dimensions 40 mm × 40 mm × 160 mm. Scanning electron microscopy (SEM) and X-ray diffraction (XRD) tests were conducted to determine the crystalline structure of geopolymers, the X-ray diffraction technique was employed using equipment from Panalytical XperPro (Malvern Panalytical, Malvern, Worcestershire, UK), with Cu-Kα radiation (wavelength λ = 1.54060 Å; voltage of 45 kV; current of 40 mA). Measurements were conducted in the upper region of the sample, covering a 2θ range from 20° to 90°. Additionally, the scanning electron microscopy with energy-dispersive spectroscopy (SEM-EDX) technique was utilized, with Tescan Vega3 model (TESCAN, Brno, Czech Republic), using secondary electrons (SE). This technique was applied to visualize and analyze the chemical composition, as well as to identify the minerals present in the geopolymers formation process. Tests were performed on samples baked at 30 °C and 60 °C. After mixing and pouring, the specimens underwent a curing process in an oven for 24 h at temperatures of 30 °C and 60 °C for the geopolymers-based mortar, while the Portland cement-based mortar cured submerged in water for the same period.

#### 2.2.1. Physical Tests

**Figure 3:**
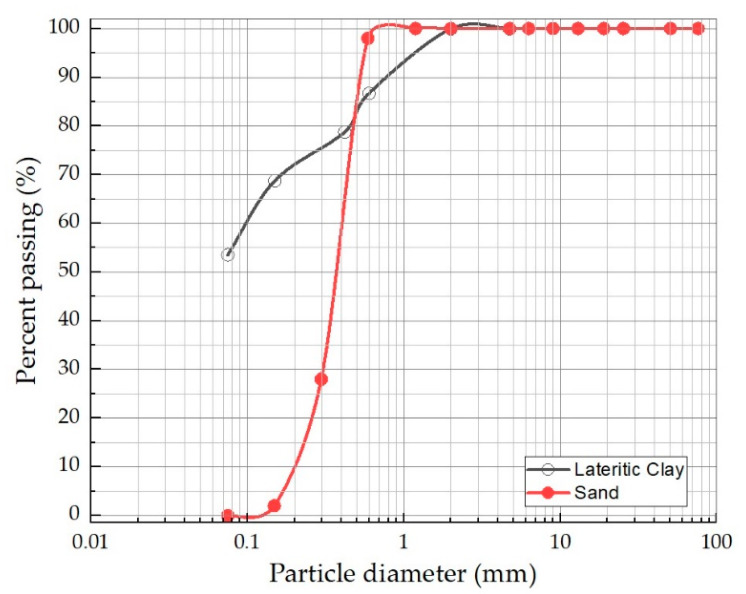
The granulometric curve of the lateritic and sand soil example.

Initially, the lateritic soil was classified as clay of low plasticity (CL) by the unified soil classification system. Next, the material added to make the mortar corresponds to sand, classified as poorly graded sand (SP), with the following characteristics: curvature coefficient of 1.47, uniformity coefficient, and specific gravity of 2.69. The characterization was carried out under ASTM D2487 criteria [19]. The results of the lateritic soil and sand particle size are shown in Figure 3. The physical properties of the lateritic soil are shown in Table 1. The lateritic sample tests were performed according to ASTM standard specifications; the standards used were raw material extraction according to the ASTM D75 method [17], determination of liquid limit, plastic limit, and plasticity index ASTM D4318 [21], total water content of aggregates by drying ASTM C566 [26], and a method for determining the true density, net density, and water absorption of fine aggregates ASTM C128 [27].

#### 2.2.2. Chemical and Mineralogical Tests

The material characterization was conducted to verify its compliance with the required chemical and mineralogical characteristics. In this process, a sieved sample of lateritic material was used, with particles passing through sieve number 200. A total of 8 kg was selected. From this quantity, 200 g was specifically extracted to carry out the initial analysis through flame photometry and chemical decomposition. Table 2 shows the result of the values presented by the material from the chemical decomposition. According to Culshaw (2001), where sodium is found to the point that it forms 10% of sodium or more of the total cation exchange capacity, the deposits are dispersible. They are prone to rapid erosion and lubrification of lateritic tropical soil. It is concluded that the material presents an amount of sodium and, therefore, is lateritic because the percentage of sodium is 0.10 and does not exceed 0.15 to consider the material a saprolitic soil [28].

Consecutively, fluorescence assay was performed, Quiniou et. al. (2014) specify that it is an assay that is used in different disciplines for chemical composition determination of a wide variety of sample types. It is nondestructive, multi-elemental, fast, and cost-effective and can be applied directly to certain types of samples without any preparation, and it is ideal for many applications [29]. As this technique combines high precision and accuracy with easy and fast sample preparation, it is possible to automate it. Likewise, it can provide both qualitative and quantitative information, making it possible to conduct a rapid detection analysis.

Thus, two samples of 200 g of the finest material passing through the sieve No. 200 were used for the test. Therefore, the test showed that these materials contain only silicon, aluminum, and iron, minerals necessary to form geopolymers. Next, with the analysis of minerals obtained through the X-ray fluorescence test, we proceeded to perform the molar ratios; for this, we worked with the atomic weights and with the weights obtained in the fluorescence and flame photometry tests. Davidovits (2013) presents molar ratio intervals and explains that a solid solution can be given starting from Si/Al = 1 (see Table 3). The same was used to validate the molar ratios to validate the geopolymer [30].

#### 2.2.3. Mechanical Testing

In the execution of mechanical tests, experiments were conducted on samples composed of lateritic clay and sand with lateritic clay. Initially, the consistency of the lateritic clay-based hydraulic binder was determined, following the normal consistency procedure established by ASTM C187 [31] for cement. The Vicat needle was allowed to drop for 30 s, permitting a penetration of at least 1 cm into the geopolymers, aiming to measure the initial setting time of the pastes.

The water-to-binder ratio was measured to assess the influence of aluminosilicate precursors used in the process, which included lateritic clay and sodium hydroxide in the formation of the hydraulic binder. A variation in the consistency of the binder was observed, attributed to the temperature increase in the mixtures of geopolymers with sodium hydroxide or clay with sand. The results of the water-to-binder ratios for pure clay and the mixture with sand are detailed in Table 4 and Table 5, respectively. This analysis enabled the identification of differences in the mechanical properties of the binder based on the composition of the mixture, contributing to a deeper understanding of its behavior and performance.

#### 2.2.4. Compression Test of 50 × 50 × 50 mm Cubes of Cement and Geopolymer Mortar

Tests were conducted on cement mortars with sand and mortars with lateritic clay-based geopolymers in accordance with ASTM C1157 [32], which adhere to the requirements outlined in ASTM C778 [31]. Initially, compressive strength tests were carried out on samples of cement and sand mortar, followed by tests on lateritic clay-based geopolymers. The cubes had dimensions of 50 mm x 50 mm, with curing times of 7, 14, and 28 days, followed by a 24 h drying period in an oven at 30 °C. The cement mortar and geopolymers were mixed in a ratio of 1:2.75, determined through molar relationships. Three samples were tested for each type of mortar (geopolymer and cement), with the average compressive strength recorded. Water–cement and water–geopolymer ratios were adjusted to achieve a fresh mix flow of 110 ± 5%, according to ASTM C1437 [32], resulting in ratios ranging between 0.40 and 0.50 for both cases.

The initial setting time of the pastes was measured using the Vicat needle apparatus. The amount of water in both mixtures, with geopolymers and cement, was measured to determine the setting time required to achieve normal consistency, following ASTM C187 [33]. Following the volume dosing relationship, it was weighted to obtain precise quantities for the preparation of cement mortar cubes. Initial quantities were as follows: cement = 82 g, sand = 226 g, and water = 60 mL for geopolymers; water and NaOH = 70 mL (alkaline solution), lateritic clay = 190 g, and sand = 118 g.

For the experimental procedure, materials (geopolymer and sand) were weighed, water was added for cement mortar, and sodium hydroxide (NaOH) was added for geopolymers. They were mixed until a homogeneous consistency was achieved, observing that the water/cement ratio was suitable for compaction. Once the mixture was prepared, the molds were filled in four layers of eight strokes, followed by smoothing the surface, and finally, the curing process was carried out.

#### 2.2.5. Bending Test for 40 × 40 × 160 mm Prisms of Cement and Geopolymer Mortars

For the prism bending test, cement mortars with sand and geopolymers based on lateritic clays were prepared. The prisms, measuring 40 mm by 40 mm by 160 mm, were fabricated with a ratio of 1:2.75, validated through molar relationships.

Tests were conducted on three samples of cement mortars with sand and geopolymers based on lateritic clays, recording the average flexural strength values. To prepare the samples of Portland cement paste and water, aimed at obtaining a mortar paste with normal consistency, and for the geopolymers, sodium hydroxide (NaOH) was added as per ASTM C187 [34]. The fresh paste was used to create prismatic samples measuring 40 mm by 40 mm by 160 mm, which were stored in sealed conditions. After 24 h, the specimens were demolded, and their initial lengths were immediately measured. Subsequently, the samples were subjected to a vapor pressure of approximately 2 ± 0.07 MPa at a temperature of 216 °C for 3 h.

The preparation of the three samples, both for cement mortar and geopolymers, followed ASTM C348 [35]. A volume dosage with a water–cement ratio of 0.485 was used, equivalent to a dosage of 1 part cement and 2.75 parts sand. It was weighted to obtain precise amounts for prism fabrication, with the following quantities: cement = 166 g, sand = 458 g, and water = 60 mL for cement mortar; for geopolymers, water and NaOH = 220 mL (alkaline solution), lateritic clay = 530 g, and sand = 168 g. These materials were carefully mixed until a homogeneous blend was achieved, followed by water addition and the continuation of the mixing process. The molds were filled starting with an initial layer of approximately 2.5 cm thickness, followed by compaction with 32 strokes on the surface in 4 stages of 8 strokes each, and finally, surface smoothing. The curing procedure was the same as previously described in the compression test section.

After the cement prisms were made, the preparation of geopolymers was initiated. In the initial phase, sodium hydroxide was mixed with distilled water in precise proportions (1 L of distilled water per 480 g of sodium hydroxide) to generate an alkaline solution. This process was conducted cautiously due to the exothermic reaction produced. The alkaline solution was poured onto the carefully combined mixture of sand and clay. Using paddles, the mixture was meticulously stirred for approximately 5 min, eliminating lumps and obtaining a homogeneous blend. Subsequently, the mixture was transferred to the molds, following the same compaction and leveling steps previously described for the cement prisms. Throughout the process, the temperature of the geopolymer was monitored. Initially, a temperature rise was observed, reaching 61 °C due to the NaOH. This thermal elevation modified the clay, raising it to 41 °C, a temperature that gradually decreased over a period of 80 min until reaching ambient temperature. It is emphasized that maintaining this material in tropical environments with temperatures above 30 degrees is crucial, as the cooling time is essential for successful geopolymerization. Hence, it is imperative to keep the material at an appropriate temperature to achieve successful geopolymerization.

**Figure 4:**
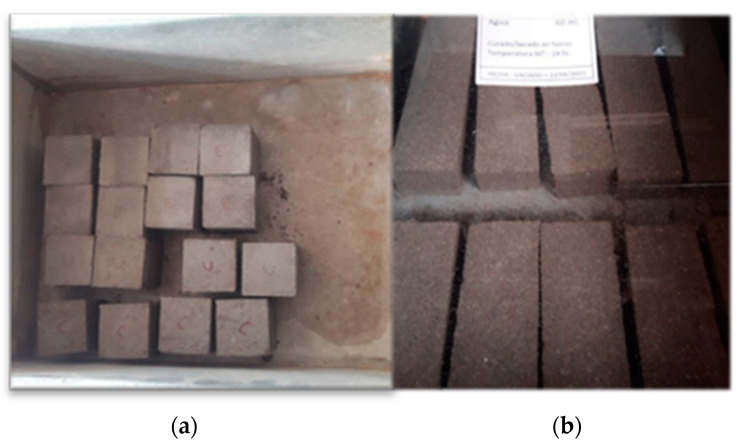
(**a**) 50 × 50 × 50 mm cement mortar cubes, (**b**) 40 × 40 × 160 mm prisms of cement mortars and geopolymers.

It is noteworthy that, in contrast to cement, which experiences a temperature rise over time, the geopolymer underwent a curing process for 24 h at a constant temperature of 30 °C, followed by exposure to a temperate environment of no less than 30 °C. Figure 4a,b, showing the cubes and prisms of cement and geopolymer mortar, are attached for visual reference.

## 3. Results and Discussion

### 3.1. Molar Ratios

With the minerals obtained by the X-ray fluorescence assay, the molar ratios were determined using the atomic weights and the weights obtained in the fluorescence and flame photometry assays. The molar ratio for the compound SiO_2_/Al_2_O_3_ is observed in Table 6.

Table 7 shows the molar ratio for the H_2_O/Na_2_O compound, and Table 8 shows the molar ratio for the Na_2_O/Al_2_O_3_ compound.

Although the range of NaOH in intervals is from 8 to 14, for the present investigation 12 moles were used. The literature recommends values up to 12 moles due to the hostile nature of NaOH (see Table 9).

Finally, for the molar ratio of the SiO_2_/NaOH the following results are shown in Table 10.

Chemical and mineralogical analyses systematically complement the mechanical analyses of material characterization and strength to evaluate the geopolymer as an alternative hydraulic binder for construction. Generally, an essential criterion for choosing laterite depends on the silica/silica oxide ratio [36]. The determination of the latter ratio, which is deduced from the chemical and mineralogical composition of the raw material, is criticized. Some authors considered total silica (SiO_2_) for its calculation, while other authors recommend considering only silica (SiO_2_) which comes mainly from aluminosilicates (the clay minerals) [36]. Considering total silica, the evaluated silica/silica oxide ratio is around 2.4. According to the literature data [36], the value obtained suggests that the materials in question could be used in construction. However, because the latter ratio is less than 2.5, the material studied needs to be improved regarding compressive strength. The use of high mechanical performance materials whose ratio is 2.5 to 6 is required.

### 3.2. Compressive Strength Test

**Figure 5:**
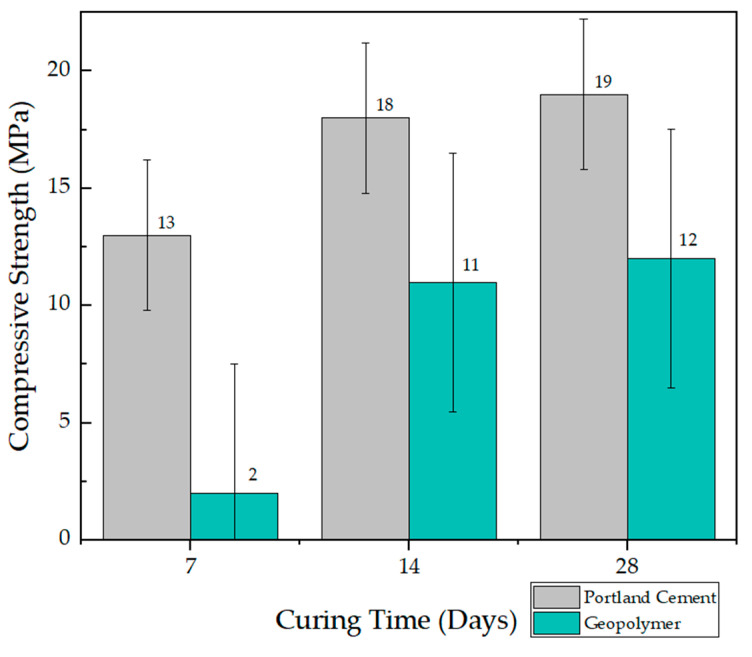
Compressive strength test results for mortar specimens after different curing periods (7, 14, and 28 days) baked at 30 °C.

**Figure 6:**
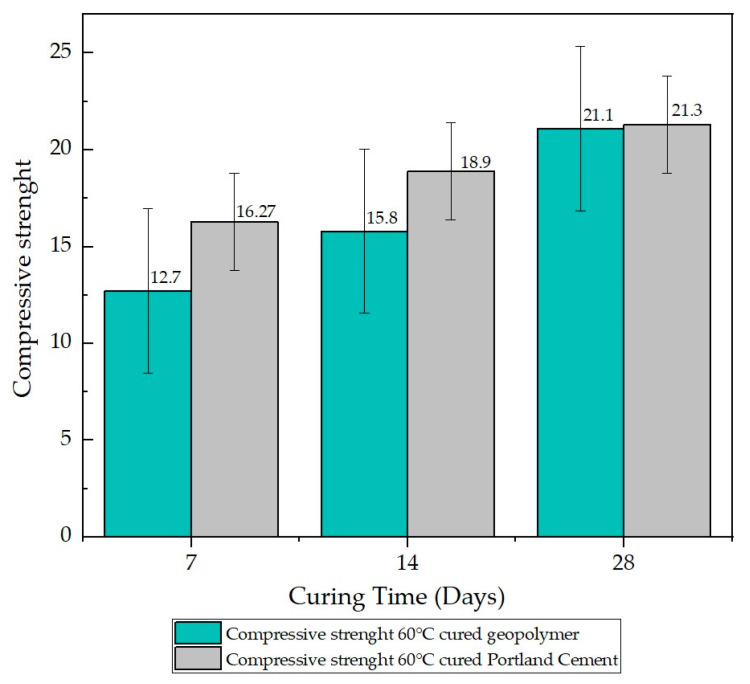
Compressive strength test results for mortar specimens after different curing periods (7, 14, and 28 days) baked at 60 °C.

The compressive strength test was used as a more accurate indicator to measure the effect of geopolymer compared to cement mortar [37]. Samples of geopolymer cubes and cement mortar were cured for 1 to 28 days to study the effect of time on their strength, for 28 days is sufficient time for the cementitious compounds in the stabilized soil to develop substantial chemical reactions [38]. The results revealed that the laterite clay-based geopolymer, compared to the mortar made with Portland cement IP, produced lower compressive strengths at all ages (up to 28 days). The results of the compressive strength tests for mortar samples prepared with Portland cement IP and the laterite clay-based geopolymer are presented in Figure 5 for samples baked at 30 °C. A visual representation is provided, highlighting the errors associated with three samples of Portland cement and three samples of geopolymers. The results reveal that the root mean square error for Portland cement samples is 3.214. In the case of geopolymers, a more pronounced root mean square error is observed, reaching a value of 5.507. These errors serve as quantitative measures assessing the variability and precision of the data, offering a detailed understanding of the consistency and reliability of the analyzed samples. Moreover, in Figure 6, the results of compressive strength for samples baked at 60 °C are presented.

In Figure 6, the error bars associated with the compressive strength sample of geopolymer cured at 60 °C and the sample of Portland cement mortar cured at 60 °C are depicted. This graph provides a detailed visual representation of the variability in the compressive strength data for these specific curing conditions. The value of the mean squared error, calculated as a quantitative measure of the discrepancy between experimental results and expected values, is recorded as 4.248 for the geopolymer and 2.516 for the Portland cement mortar sample. These figures underscore the magnitude of uncertainty associated with the compressive strength measurements in both samples cured at 60 °C, offering a deeper assessment of the reliability of these results.

The results of the compression tests have determined that the mechanical behavior of the geopolymer, in terms of compressive strength, is 64% less compared to Portland cement IP; therefore, the geopolymer based on laterite clays does not meet the compressive strength to match cement. It is also observed that the longer the curing time for the Portland cement mortar, the more significant the increase in strength, from 13 to 19 MPa. This indicates that the cementitious reactions continued to develop throughout the curing period. The studies of Baldovino et al. [39] have also shown a higher strength of cementitious mixes for extended periods. This trend is similar to studies that stated that the compressive strength of cubes is lower in strength compared to cement [14].

The compression results presented are related to various studies conducted worldwide, where compression strengths ranged between 2 to 10 MPa, respectively, with samples from Ivory Coast, Cameroon, and Ghana. This is due to the high clay percentage in these lateritic gravels compared to the studied sample in this study. Overall, clays contribute to the reduction in material compression strength due to their plasticity [23].

The geotechnical properties of the studied sample are generally better than those reported for lateritic clays in Ghana and Ivory Coast [36]. The lateritic clays in these countries contain a high content of fines primarily composed of clay minerals compared to the studied sample. It is well known that clay minerals have detrimental effects on geotechnical properties. The observed differences are likely attributed to differences in the genesis of laterite soils and also the climate. In fact, laterite soils in Ghana and Ivory Coast form in humid climates with significant annual rainfall, while those in Bolivia develop in tropical climates with precipitation occurring at specific times of the year [23].

### 3.3. Bending Test

**Figure 7:**
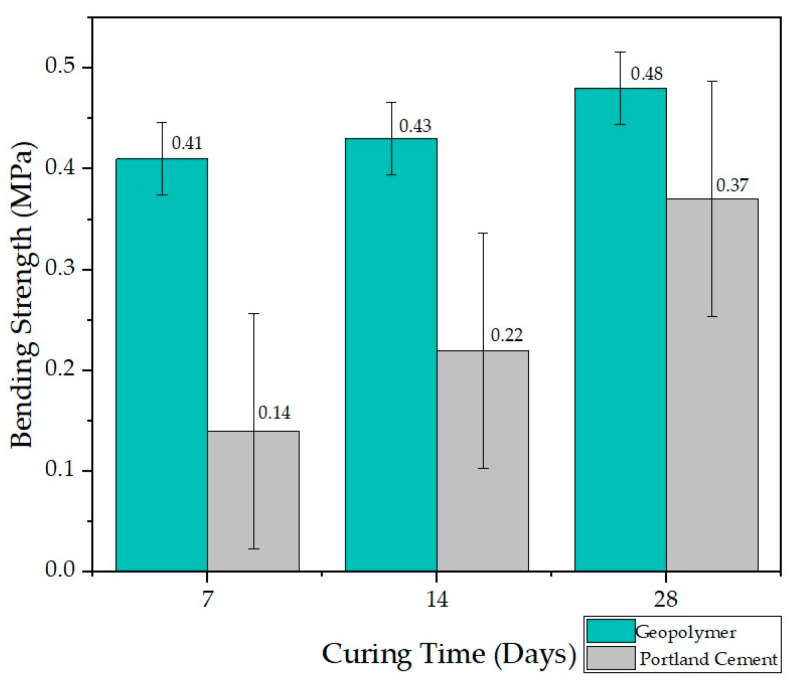
Bending test results for mortar specimens after different curing periods (7, 14, and 28 days).

**Figure 8:**
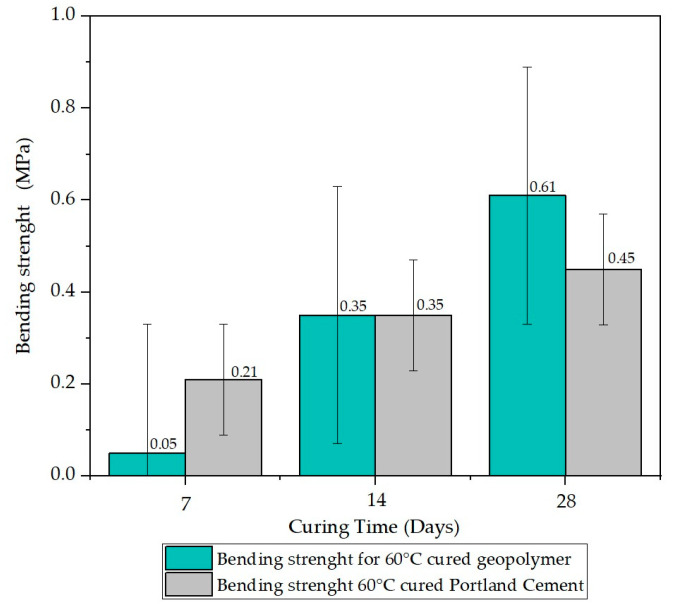
Bending test results for mortar specimens after different curing periods (7, 14, and 28 days) for samples baked at 60 °C.

Within the set of experiments, the strength of mortar and geopolymer beams was experimentally evaluated. The beam specimens were tested under point loading at mid-span with an active test span of 650 mm (distance between the ends of both supports). The beam specimens were slowly loaded to failure. The resistance to flexural failure obtained from the maximum sustained load before failure of the test beam is measured for strength [4]. The flexural strength tests in Figure 7 show that the geopolymer outperforms flexural strength by 29.3% compared to the Portland cement IP mortar beam. The visual representation of the results highlights the inclusion of error bars in the assessment of flexural test for three samples of Portland cement and geopolymers. This graphical representation provides a more comprehensive perspective on the variability in experimental results, enhancing the understanding of data dispersion and allowing for a visualization of the magnitude of uncertainty associated with each test set. The errors associated with Portland cement show a mean value of 0.116, whereas for geopolymers, the mean error is 0.036. In this sense, in Figure 8, is presented the results of bending strengths for samples baked at 60 °C. In the analysis of the flexural tests conducted during the 60 °C curing process for both the geopolymer and Portland cement mortar, the respective mean squared errors were obtained. For the geopolymer, a value of 0.280 was recorded, while for the Portland cement mortar, the mean squared error was 0.121. These figures reflect the inherent uncertainty in the data obtained during the flexural tests, providing a quantitative perspective on the variability and precision associated with the results of these experiments.

### 3.4. X-ray Diffraction and Scanning Electron Microscopy (SEM)

The X-ray diffraction test required 20 g of two samples of the geopolymer. The test in the X-ray machine allows for obtaining the incoming and outgoing peaks of the beam in the material. The peak list is shown in Table 11.

As a result of the reading, it is possible to know the compounds obtained after the geopolymerization between sodium hydroxide and aluminosilicates. These chemicals are presented in Table 12.

Two fragments of solid samples were analyzed by scanning electron microscopy with energy-dispersive X-ray spectroscopy (SEM-EDX). Scanning electron microscopy was performed on two samples baked at 30 °C and 60 °C. The objective of the analysis was to obtain surface micrographs at magnifications of 500×, 1 k×, and 7 k×.

**Figure 9:**
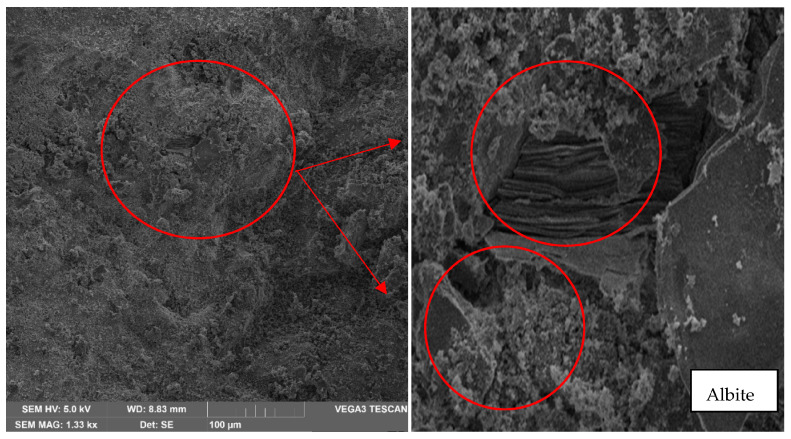
Analysis result of sample 1 in oven at 30 °C—500× magnification—Albite presence.

Next, it can be observed that the first sample baked at 30 °C, with a zoom of 500×, shows Albite formations, identified by the irregular to colloidal shape and folds, as shown in Figure 9.

**Figure 10:**
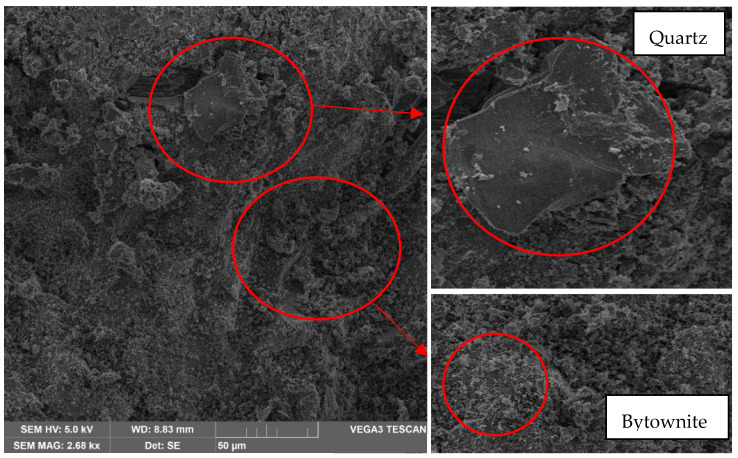
Analysis result of sample 1 in oven at 30 °C—500× magnification—Presence of quartz and Bytownite.

From a 500× magnification, which is the lowest, the albite content can be appreciated. This material belongs to the sodium plagioclase family, which is an aluminosilicate. It presents a hardness of 6–6.5 Mohr on a scale of 1 to 10 hardness, so it is considered a material with high hardness and lightness. Moreover, as shown in Figure 10, the presence of quartz, a smooth material of irregular structure with colloidal edges, has a hardness of 7 Mohr on a scale of 1 to 10. In Figure 10, it can also be observed the presence of Bytownite, a material that presents an irregular structure similar to clay lumps; this material is part of the feldspars, with a hardness of 6–6.5 Mohr on a scale from 1 to 10.

**Figure 11:**
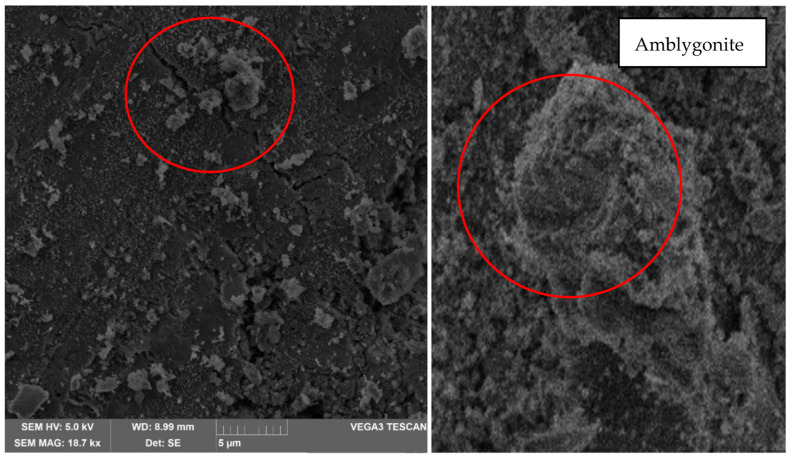
Analysis result of sample 1 in oven at 30 °C—7 k× magnification—Presence of amblygonite and fractures.

In addition, in Figure 11, the presence of amblygonite can be identified by the irregular subconchoidal shape since its faces are convex on one side and concave on the other. This material has a hardness of 5.5 to 6 Mohr on a scale of 1 to 10. It should be noted that all these appreciations were achieved with a magnification of 500× and 7 k×; however, it can be observed at 18.7 k× (red circle in Figure 11) that fractures up to 10 µm were formed. Although a geopolymerization has been performed, the degree of alloy between minerals is not high enough to reduce the fracture and that there is no union between the different minerals in their entirety.

**Figure 12:**
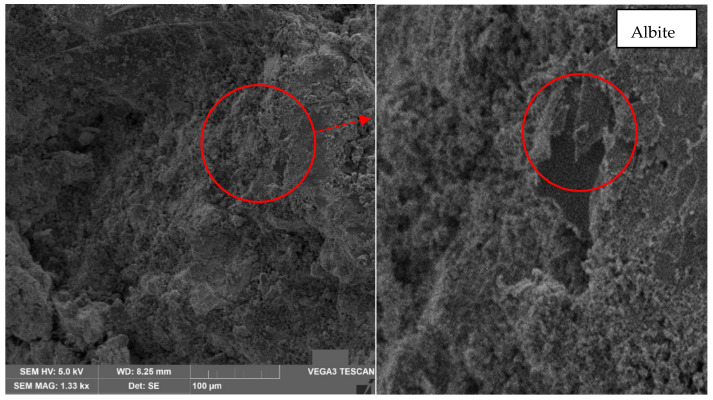
Analysis result of sample 2 in oven at 60 °C—500× magnification—Presence of albite.

As for the second sample in the oven at 60 °C, albite (circles in red) is observed at a magnification of 500× (Figure 12) due to the colloidal surface. However, this is not as noticeable as in the 30 °C sample.

**Figure 13:**
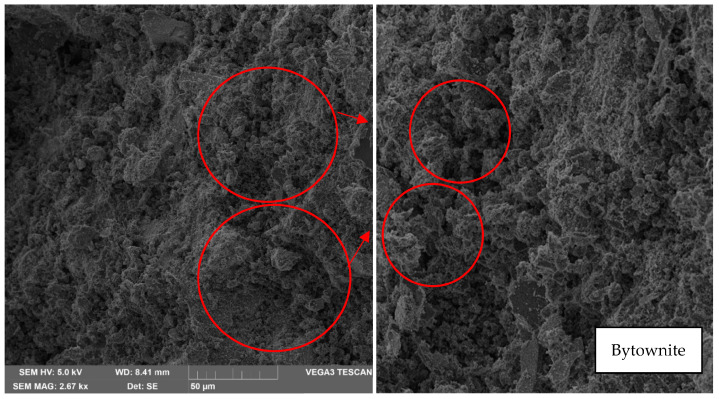
Analysis result of sample 2 in oven at 60 °C—1 k× magnification—Presence of bytownite.

Likewise, a high content of Bytownite can be observed highlighted in circle in red due to its irregular shape as united lumps. This material has a higher feldspar content (Figure 13).

**Figure 14:**
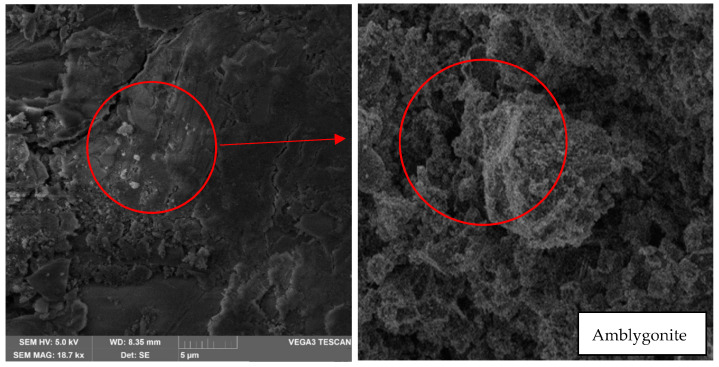
Analysis result of sample 2 in oven at 60 °C—7 k× magnification—Presence of amblygonite.

On the other hand, in Figure 14, it can be observed that it contains amblygonite (circles in red) due to the irregular subconchoidal shape. Likewise, in Figure 14, a clear difference can be seen with an increase of 7 k× since it is possible to observe that the sample has a fracture of 0.25 µm, which indicates that due to the temperature, the material has a better degree of polymerization since the minerals have a better alloy between them.

**Figure 15:**
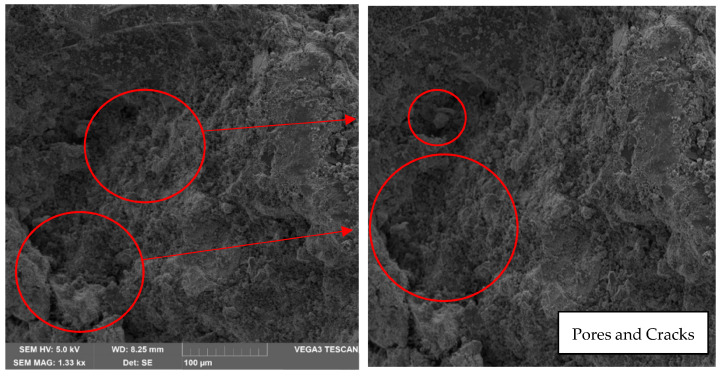
Analysis result of sample 2 in oven at 60 °C—1 k× magnification—Presence of voids.

On the other hand, an increase in pores, voids and fractures can be evidenced in the sample that was manufactured at 60 °C, as shown in Figure 15 in circles in red.

The microstructures observed in the SEM micrographs provided in the preceding figures reveal the combination of well-developed pores and cavities, which can form due to water evaporation during curing and the geopolymerization process [29,36,40]. The presence of these pores and the formation of various crystals on the surface are crucial for understanding the behavior of the lateritic clay-based geopolymer. In this sense, all geopolymer samples underwent on compressive strength tests after curing for 7, 14, and 28 days, and the results are summarized in Figure 5 and Figure 6. Additionally, Figure 7 and Figure 8 display the results of the bending test.

The concentration of different components has a significant effect on the mechanical strengths of laterite-based geopolymers. Higher strengths were achieved in geopolymers cured for 28 days and baked at 60 °C. This trend aligns with previous studies [9,10,14,40,41], demonstrating that compression strength increases with the molar concentration of NaOH. Hence, these samples exhibit a high degree of hydrolysis and dissolution of silicon, iron, and aluminum, containing species that polymerize/condense to form geopolymers with higher strength [41,42]. This translates into the presence of quartz, albite, and bytownite, contributing to the improvement of compression and flexural strength in the tested samples, as observed in other studies globally [5,12,13,41,42,43]. Although the compression strengths are lower compared to Portland cement, they are suitable for applications not requiring higher strength.

## 4. Conclusions

The overall objective of this study is to evaluate the compressive strength and microstructural characteristics of a geopolymer based on laterite clays. The study has among its objectives to complete the knowledge gaps on alternative binder materials with low emissions of CO_2_. Therefore, it is necessary to highlight that, in this first phase, mechanical, chemical, and mineralogical aspects were evaluated with the geopolymer, which gave positive results of the geopolymer as an alternative to be used, allowing this to be deepened in other studies.

Through the results of mechanical strength and microstructure, it is possible to determine that the mechanical behavior of the geopolymer, in terms of compressive strength, is 64% compared to the Portland cement IP. Thus, the geopolymer based on lateritic clays does not meet the compressive strength to match cement. However, using scanning electron microscopy and the X-ray scattering test, it can be determined that the decrease in compressive strength is because constant temperatures from 30 °C are required in a controlled environment to avoid the generation of voids and generate better bonds between minerals due to the presence of plagioclases and feldspars that are also found in Portland cement and that with a better alloy, a higher compressive strength will be generated. On the other hand, the results of the flexural strength tests determine that the geopolymer is outperforming in flexural strength by 29.3% compared to Portland cement IP.

The scanning electron microscopy indicates the presence of albite. This material belongs to the sodium plagioclase family, which is an aluminosilicate; quartz, a smooth material of irregular structure with colloidal edges; bytownite, a material that presents an irregular structure similar to clay lumps and the presence of amblygonite, all of them provide hardness to the material. However, it is essential to highlight that the fractures are because, although a geopolymerization has been performed, the degree of alloy between minerals is not high enough to reduce the fracture and that there is no union between the different minerals in their entirety.

It is possible to establish that the higher the degree of geopolymerization, the greater the mechanical strength, as evidenced by the results of X-ray diffraction and scanning electron microscopy, and that the higher the amount of heat, the better the bonds are generated. However, due to continuous uncontrolled exposure, it expands with the heat, generating air voids that turn the material into one with lower resistance, so it is recommended to have the material under constant control at the time of curing.

## Figures and Tables

**Table 1:** Properties of lateritic soil.

Properties	Method	Lateritic Soil
Plastic index, %	ASTM [21]	15.96
Plastic limit, %		20.83
Liquid limit, %		36.8
USCS classification	ASTM [19]	CL

**Table 2:** Flame photometry, chemical decomposition.

Name	Nomenclature	Materials (%)
Aluminum	Al	0.50
Iron	Fe	14.08
Sodium	Na	0.10
Silica	SiO_2_	52.70
Aluminum Oxide	Al_2_O_3_	0.95
Iron Oxide	Fe_2_O_3_	20.13

**Table 3:** Molar relations according to Davidovits [30].

Molar Relations	Intervals
SiO_2_/Al_2_O_3_	1–5
H_2_O/Na_2_0	10–25
Na_2_O/Al_2_O_3_	0.1–0.3
NaOH	8–14
SiO_2_/NaOH	0–3

**Table 4:** Vicat needle test with clay.

Lateritic Clay		
Time (s)	Relation a/c	Needle Penetration (cm)
1	1.88	4.00
3	1.40	3.00
6	1.04	1.50
15	0.92	1.20
30	0.80	0.80

**Table 5:** Vicat’s needle test with clay and sand.

Lateritic Clay with Sand		
Time (s)	Relation a/c	Needle Penetration (cm)
10	1.00	1.50
15	0.80	1.20
20	0.75	1.10
30	0.70	0.90
30	0.60	0.70

**Table 6:** Molar relations of SiO_2_/Al_2_O_3_.

SiO_2_/Al_2_O_3_				
Element	Weight per 200 g	Specific Weight of the Element g/mol	Moles	Molar Relation
SiO_2_	52.71	60.08	0.877	2.4
Al_2_O_3_	37.43	101.96	0.367	

**Table 7:** Molar relations of H_2_O/Na_2_O.

H_2_O/Na_2_O				
Element	Weight per 200 g	Specific Weight of the Element g/mol	Moles	Molar Relation
H_2_O	0.49	18.02	0.03	12.84
Na_2_O	0.13	61.98	0.002	

**Table 8:** Molar relations of Na_2_O/Al_2_O_3_.

Na_2_O/Al_2_O_3_				
Element	Weight per 200 g	Specific Weight of the Element g/mol	Moles	Molar Relation
Na_2_O	0.13	61.98	0.002	0.006
Al_2_O_3_	37.43	101.96	0.367	

**Table 9:** Molar relations according of NaOH.

Moles	Specific Weight of the Element g/mol	Weight of NaOH per Liter of Water
6	39.997	239.982
8	39.997	319.976
12	39.997	479.964
14	39.997	559.958

**Table 10:** Molar relations of SiO_2_/NaOH.

SiO_2_/NaOH				
Element	Weight per 200 g	Specific Weight of the Element g/mol	Moles	Molar Relation
SiO_2_	52.70	60.080	0.877	0.75
NaOH	46.57	39.997	1.164	

**Table 11:** Peak list of X-ray diffraction test.

Position 2θ [°]	Height [cts]: Peaks Height	Full Width at Half Maximum Left [2θ.]	d-Spacing [Å]: Spacing between Crystal Lattice Planes in a Crystalline Material	Relative Intensity [%]
20.889170	135.113900	0.078720	4.25264	16.02
22.077390	63.912460	0.098400	4.02637	7.58
23.099600	23.187560	0.157440	3.85046	2.75
23.550370	59.703910	0.098400	3.77777	7.08
24.306640	58.254090	0.236160	3.66191	6.91
25.517900	15.102580	0.236160	3.49078	1.79
26.660950	843.196500	0.137760	3.34365	100.00
27.477370	148.675300	0.078720	3.24613	17.63
27.957560	693.256500	0.098400	3.19146	82.22
29.475250	12.510900	0.118080	3.03049	1.48
30.182550	45.439030	0.157440	2.96107	5.39
31.264770	23.343700	0.118080	2.86100	2.77
35.012180	33.909410	0.196800	2.56289	4.02
36.570580	97.058420	0.059040	2.45718	11.51
37.743020	9.881138	0.472320	2.38350	1.17
39.510780	35.749520	0.118080	2.28084	4.24
40.346860	26.344840	0.157440	2.23549	3.12
42.483920	43.592150	0.157440	2.12785	5.17
45.819530	39.157040	0.157440	1.98041	4.64
48.142280	12.551990	0.314880	1.89015	1.49
50.167770	163.465200	0.078720	1.81849	19.39
53.304990	6.532775	0.236160	1.71862	0.77
54.964550	13.563550	0.236160	1.67060	1.61
59.991550	56.058900	0.078720	1.54207	6.65
63.828960	6.545800	0.629760	1.45830	0.78
67.759100	32.907390	0.118080	1.38297	3.90
68.352640	54.820680	0.096000	1.37127	6.50
75.751880	10.211800	0.236160	1.25569	1.21
77.838260	8.107481	0.472320	1.22717	0.96
79.995570	8.126995	0.393600	1.19942	0.96
81.460400	9.188509	0.472320	1.18152	1.09

**Table 12:** Compounds obtained after the geopolymerization.

Ref. Code	Compound Name	Chemical Formula
96-500-0036	Quartz	Si_3_O_6_
96-900-9664	Albite	Na_1.96_ Ca_0.04_ Si_5.96_ A_12.04_ O_16_
96-901-1202	Bytownite	Ca_6.88_ Na_1.12_ Al_15.52_ Si_16.48_ O_64_
96-900-2880	Amblygonite	P_2_ Al_2_ O_8.88_ F_1.08_ H_0.88_ Li_2_

## Data Availability

Data are contained within the article.
